# A speech fluency brain network derived from gliomas

**DOI:** 10.1093/braincomms/fcae153

**Published:** 2024-04-29

**Authors:** Cechen Sun, Jie Zhang, Linghao Bu, Junfeng Lu, Ye Yao, Jinsong Wu

**Affiliations:** Department of Biostatistics, School of Public Health & National Clinical Research Centre for Aging and Medicine, Huashan Hospital, Fudan University, Shanghai 200040, China; Department of Neurosurgery, Huashan Hospital, Shanghai Medical College, Fudan University, Shanghai 200040, China; National Center for Neurological Disorders, Shanghai 201107, China; Shanghai Key Laboratory of Brain Function Restoration and Neural Regeneration, Shanghai 200040, China; Neurosurgical Institute of Fudan University, Shanghai 200040, China; Shanghai Clinical Medical Center of Neurosurgery, Shanghai 200040, China; Department of Neurosurgery, Huashan Hospital, Shanghai Medical College, Fudan University, Shanghai 200040, China; National Center for Neurological Disorders, Shanghai 201107, China; Shanghai Key Laboratory of Brain Function Restoration and Neural Regeneration, Shanghai 200040, China; Neurosurgical Institute of Fudan University, Shanghai 200040, China; Shanghai Clinical Medical Center of Neurosurgery, Shanghai 200040, China; Department of Neurosurgery, Huashan Hospital, Shanghai Medical College, Fudan University, Shanghai 200040, China; National Center for Neurological Disorders, Shanghai 201107, China; Shanghai Key Laboratory of Brain Function Restoration and Neural Regeneration, Shanghai 200040, China; Neurosurgical Institute of Fudan University, Shanghai 200040, China; Shanghai Clinical Medical Center of Neurosurgery, Shanghai 200040, China; Department of Biostatistics, School of Public Health & National Clinical Research Centre for Aging and Medicine, Huashan Hospital, Fudan University, Shanghai 200040, China; Key Laboratory of Public Health Safety of Ministry of Education, Fudan University, Shanghai 200032, China; Department of Neurosurgery, Huashan Hospital, Shanghai Medical College, Fudan University, Shanghai 200040, China; National Center for Neurological Disorders, Shanghai 201107, China; Shanghai Key Laboratory of Brain Function Restoration and Neural Regeneration, Shanghai 200040, China; Neurosurgical Institute of Fudan University, Shanghai 200040, China; Shanghai Clinical Medical Center of Neurosurgery, Shanghai 200040, China

**Keywords:** speech fluency, low-grade glioma, language network

## Abstract

The brain network of speech fluency has not yet been investigated via a study with a large and homogenous sample. This study analysed multimodal imaging data from 115 patients with low-grade glioma to explore the brain network of speech fluency. We applied voxel-based lesion-symptom mapping to identify domain-specific regions and white matter pathways associated with speech fluency. Direct cortical stimulation validated the domain-specific regions intra-operatively. We then performed connectivity-behaviour analysis with the aim of identifying connections that significantly correlated with speech fluency. Voxel-based lesion-symptom mapping analysis showed that damage to domain-specific regions (the middle frontal gyrus, the precentral gyrus, the orbital part of inferior frontal gyrus and the insula) and white matter pathways (corticospinal fasciculus, internal capsule, arcuate fasciculus, uncinate fasciculus, frontal aslant tract) are associated with reduced speech fluency. Furthermore, we identified connections emanating from these domain-specific regions that exhibited significant correlations with speech fluency. These findings illuminate the interaction between domain-specific regions and 17 domain-general regions—encompassing the superior frontal gyrus, middle frontal gyrus, inferior frontal gyrus and rolandic operculum, superior temporal gyrus, temporal pole, inferior temporal pole, middle cingulate gyrus, supramarginal gyrus, fusiform gyrus, inferior parietal lobe, as well as subcortical structures such as thalamus—implicating their collective role in supporting fluent speech. Our detailed mapping of the speech fluency network offers a strategic foundation for clinicians to safeguard language function during the surgical intervention for brain tumours.

## Teaser

Sun et.al investigate the brain network of behavioral measures of speech fluency in a relatively large (*n* = 115) cohort of low-grade gliomas, with additional analysis comparing intra-operative stimulation mapping. Results showed that speech fluency engages a bilateral frontal-temporal-parietal-limbic network supported by four domain-specific regions and 17 domain-general regions.

## Graphical Abstract

**Graphical Abstract:**
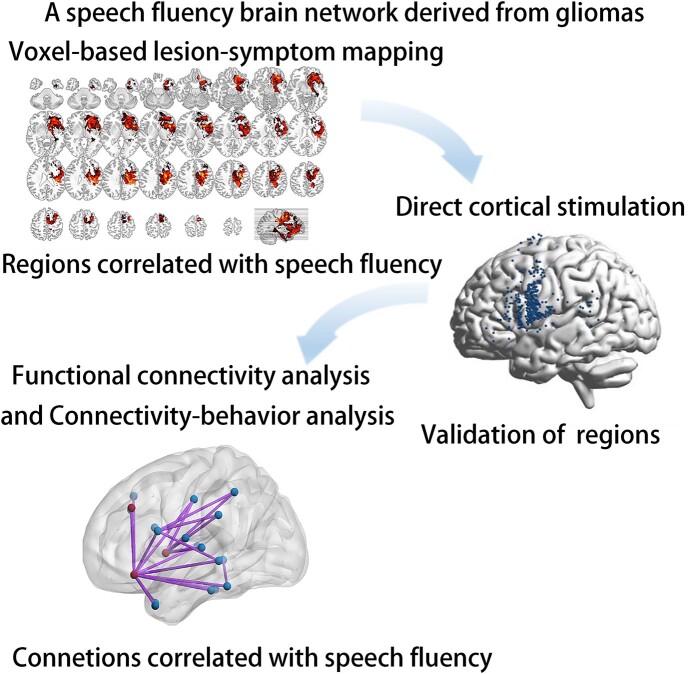

## Introduction

Speech fluency is a multifaceted function that involves word retrieval from language-specific information in memory, turning words into sentences with grammatical formulation, and motor speech production to produce fluent speech.^1^ The inability to speak fluently can greatly compromise the social interaction of patients. Apart from disrupting a person’s ability to communicate effectively, the personality characteristics of non-fluent speakers tend to be rated less favourable than those of fluent speakers.^2^ Clinically, speech fluency is increasingly viewed as an index to determine the aphasia type and its severity. Both the Western Aphasia Battery (WAB) and the Boston Diagnostic Aphasia Exam (BDAE) incorporate tasks to measure speech fluency performance.^3,4^ While the behavioural assessment of speech fluency is widely recognized, the neural underpinnings remain not fully understood. The classical Wernicke-Lichtheim-Geschwind (WLG) language model indicated that impaired speech fluency is associated with lesioned Broca’s area. Over the past two decades, MRI studies further located various brain regions responsible for speech fluency other than Broca’s area, such as the anterior insula, the precentral gyrus and the premotor cortex.^5,6^

Increasing evidence from diffusion tensor imaging (DTI) studies showed that damage to deep white matter tracts such as frontal aslant tract and arcuate fasciculus also has negative impacts on speech fluency.^6-8^ These results suggested that rather than a confined cortical region, a more widely distributed network associated with speech fluency may exist. However, the brain network of speech fluency, which refers to the combination of brain regions and the connectivity associated with speech fluency has not yet been investigated via a cohort study with a large and homogenous sample.^8,9^ Especially for brain tumour patients, a better knowledge of the brain network of speech fluency before surgery is essential to achieve maximal safe resection, thus minimizing postoperative language deficits.

Fedorenko *et al.*^10^ once proposed the idea that the two-components language network, one component includes ‘core’ regions that functionally specialized for language processing, the other includes domain-general ‘support’ regions, which may coactivate with the language core regions sometimes, but with other specialized systems at other times. Accordingly, we hypothesized that fluent speech engages a brain network consisting of two components: the ‘domain-specific’ regions of the left hemisphere that functionally specialized for speech fluency and ‘domain-general’ regions of both left and right hemisphere that functionally interact with the domain-specific regions when fluent speech occurs. Domain-specific and domain-general regions are connected by underlying white matter tracts. To test this hypothesis, we used multimodal imaging to investigate the brain network of language fluency in a cohort of low-grade gliomas (LGGs) (*N* = 115). LGGs are slow-growing, widely distributed brain tumours that can cause structural damage to the brain network.^11^ First, we used voxel-based lesion-symptom mapping (VLSM) to identify domain-specific regions associated with speech fluency as well as white matter pathways. We employed direct cortical stimulation (DCS) known as the gold standard of intraoperative brain language mapping to validate the domain-specific brain regions. Second, we calculated functional connectivity at the whole-brain level and performed a connectivity-behaviour analysis to identify connections around the domain-specific regions that significantly correlated with speech fluency. Although tumour growth may trigger underlying reorganizations, our results from a relatively large cohort (115) provide novel insight into the brain network involved in speech fluency. Moreover, by providing direct evidence in patients with glioma, our findings can guide clinicians to accurately protect speech fluency function during the resection of brain tumours.

## Materials and methods

### Participants

Participants were recruited from the Glioma Surgery Division in the Neurological Surgery Department at Huashan Hospital, Shanghai Medical College, Fudan University, Shanghai, China. Preoperative evaluations were conducted one day before surgery for all participants. Inclusion criteria included the following: native Chinese speakers between the ages of 18 and 65, right-handed as determined by the Edinburgh Handedness Inventory (EHI),^12^ and had been diagnosed pathologically with low-grade glioma (WHO grade II) in the left frontal, temporal, parietal, or insular lobes within the past five years. Patients of repeated surgeries were excluded. Patients with cognitive disorders (Mini-Mental State Examination [MMSE] < 14) were also excluded. Additionally, an experienced neuropsychologist (Y. Zhou) differentiated patients with dysarthria from those with aphasia, based on their ability to move mouth, lips, tongue and to breathe. Consequently, the current study excluded patients diagnosed with dysarthria. Patients with severe language deficits (AQ < 50) were also excluded. All patients provided written informed consent for the procedure, and the study was approved by the Institutional Review Board of Huashan Hospital.

### Clinical and behavioural characteristics

Clinical and behavioural information on all included patients was collected. The maximum diameter of each tumour, defined as the diameter of the bounding sphere that fully enclosed the tumour, and tumour volume were measured (determined on the MNI space). All patients were evaluated by the 30-point MMSE, which was utilized to exclude patients with severe cognitive disorders. Functional status was evaluated by the 100-point Karnofsky performance scale (KPS). Speech fluency was assessed using the Aphasia Battery of Chinese (ABC) one day prior to surgery. ABC is the Chinese standardized adaptation of the Western Aphasia Battery (WAB),^13^ ABC has been widely used in previous studies of Chinese native speakers and recommended by Chinese linguistic experts.^14-19^ ABC speech fluency was assessed with two tasks, the ‘conversational interview’ task and the ‘description’ task (Supplementary Fig. 1). The ‘conversational interview’ task contains six routine questions (e.g. questions such as ‘how are you today?’, ‘have you been here before?’, ‘what is your name?’, ‘what is your occupation?’, and ‘what is your address?’). The ‘description’ task comprises two subtasks: ‘picture description’ and ‘disease description’. During the Picture Description task, the examiner presents two images and instructs: ‘Please describe what you observe in these pictures, making an effort to use complete sentences.’ One picture includes a woman and a dog, another picture is the classic ‘cookie theft’ picture.^20^ In disease description task, participants were asked one open-ended question: ‘tell me a little about why you are here? or what seems to be the trouble?’. During the assessment, each participant’s fluency was evaluated by an experienced neuropsychologist using a scale of 0–10. The scoring criteria for the ABC fluency scale can be found in Supplementary Table 1. Each score corresponds to a description of speech fluency, from short, telegraphic utterances, speech output characterized by hesitations, to fluent speech characterized by normal syntactic structure.

### Imaging acquisition

MRI data were obtained in the diagnostic room of an MRI-integrated neurosurgical suite (IMRIS, Winnipeg, Canada) using a 3.0T scanner (MAGNETOM Verio 3.0T, Siemens AG, Erlangen, Germany) one day prior to the surgery for each participant. A fluid-attenuated inversion recovery (FLAIR) sequence was used for structural imaging (TR, 9000 ms; TE, 96 ms; TI, 2500 ms; flip angle, 150 degrees; slice thickness, 2 mm; FOV, 240 × 240 mm^2^; matrix size, 256 × 160; voxel size, 1 × 1 × 1 mm). For resting-state fMRI, the recorded time was 480 sec and fMRI scans were obtained every 2 sec with the subjects instructed to relax and keep their eyes open, without the need to focus on any task. Thus, the data consist of 240 time points. Dual-echo gradient-echo echo-planar images (GE-EPI) were acquired to enhance sensitivity, using an 8-channel SENSE head coil (SENSE factor 2, anterior-posterior direction, short TE = 12 ms, long TE = 35 ms, flip angle = 85°, FOV = 255 × 255 mm^2^, in-plane resolution = 3 mm × 3 mm, slice thickness = 4 mm, TR = 2000ms, 40 descending axial slices, 240 time points per acquisition). Scans were inspected immediately after each acquisition, and if motion was detected, scans were repeated.

### Imaging pre-processing

Using MRIcron software, tumour lesions were delineated from T2-FLAIR images by two neurosurgeons (J. Zhang and L. Bu), who were blinded to the behavioural data. The tumours were identified as the hyper-intense portion of the T2-FLAIR images, as described by the Response Assessment in Neuro-Oncology (RANO) criteria.^21^

All images were pre-processed using Statistics Parametric Mapping 12 (https://www.fil.ion.ucl.ac.uk/spm/software/spm12/). Structural imaging data in patients with gliomas were pre-processed by application of an optimized cost function masking with the tumour lesion masks to normalize the lesioned brain into a standard Montreal Neurological Institute (MNI) template on a 1 × 1 × 1 mm^3^ scale.^22^ All resulting normalized images were visually inspected by a senior neurosurgeon (J. S. Wu), who compared these images with the original lesions to ensure that the procedure had been performed correctly. The overlap of patient lesions is shown in Supplementary Fig. 2.

For functional image data preprocessing in patients with gliomas, weighted summation of the dual-echo images produced a single set of low-artifact functional images. The images were subjected to retrospective physiological correction. The first ten volumes of fMRI datasets were discarded, to allow for scanner stabilization, with further processing performed using spm12. After slice-timing correction and realignment to the middle volume, the functional scans were spatially normalized using the unified segmentation approach to a standard template (Montreal Neurological Institute) and resampled to 3 × 3 × 3 mm^3^. The data were smoothed using a Gaussian kernel of 8 mm full-width at half-maximum, detrended and passed through a band-pass filtre (0.01–0.08 Hz) to reduce low-frequency drift and high-frequency physiological noise. Nuisance covariates, including head motion, white matter signals and cerebrospinal signals, were regressed out from the time series. Regional time series were extracted in each of the 90 AAL-based brain regions by averaging the signals of all voxels within a region.

### Direct cortical stimulation (DCS)

Direct cortical stimulation (DCS), also called direct electrical stimulation (DES), has been used extensively in brain language mapping.^23,24^ For all patients, a 5-mm-wide bipolar electrode stimulator (Epoch XP, Axon Systems) was used to deliver a biphasic square-wave pulse at 60 Hz. The recording needle electrodes were implanted in the patient’s scalp, tendon and muscle belly of the contralateral target muscles to record compound muscle action potential. Another 4- or 6-contact strip subdural electrode was used to record after-discharge activity. The mapping procedure started from a minimum stimulation current of 1 mA and slowly increased to a maximum of 6 mA. The threshold of stimulation current would decrease by 0.5–1 mA when detecting the after–discharge potentials. This threshold was then adopted as the upper limit for the sequential stimulation. The same range of cortex was exposed after frontotemporal craniotomy in all patients (Supplementary Fig. 3). The exposed cortex was stimulated at an interval of 1 cm, during which participants were instructed to perform number counting. Speech arrest was identified when counting is completely interrupted without obvious oral, facial, mandibular and laryngeal muscle movement. Each site was discontinuously stimulated at least three times and was determined as a positive site when at least two of them induced speech arrest. Finally, to assess the spatial distribution of speech arrest sites of all patients, the speech arrest sites were marked on the surface of the brain with sterile tags, and the locations were recorded with photographs and navigational snapshots.

### VLSM analysis

The relationship between glioma lesions and speech fluency was analysed by VLSM on a voxel-by-voxel basis to identify domain-specific regions involved in speech fluency. In the VLSM analysis, two-sample *t*-test was done to compare the speech fluency scores in individuals with a lesion versus individuals without a lesion in each voxel. This test was repeated for every voxel, yielding a set of *t*-values. In other words, in each voxel, a linear regression was run with lesion status as the independent variable and speech fluency scores as the dependent variable. Moreover, we added covariates (age, sex and years of education) in the regression analysis to remove their effects. For multiple comparisons correction, we used cluster-level family-wise error correction via permutation testing, which randomly re-pairs the lesioned voxels with behavioural scores and re-runs the *t*-tests across all voxels. This test was repeated 1000 times resulting in a distribution of the *t*-values, which was used to determine the cut-off score at *P* < 0.05. Voxels were overlaid on the Natbrainlab and AAL atlases.^22,25^ VLSM analysis was performed using the publicly available VLSM toolbox (https://aphasialab.org/vlsm/) based on MATLAB software (2019b, MathWorks Inc.). To validate the results from VLSM analyses, we used an exact (significance) test to examine the relationship between VLSM voxels and sites of DCS. The effects of age, sex and years of education were removed as covariables.

### Brain network analysis

For brain network analysis, we extracted time series by averaging all voxels composing each region for each time point from 90 regions of the Anatomical Automatic Labeling (AAL) atlas. We calculated the Pearson correlation coefficients between each pair of regions. Next, 115 individual functional connectivity matrices of 90 × 90 were constructed. Addressing the multiple comparisons issue in our functional connectivity analysis, we implemented the Network-Based Statistic (NBS) method to regulate the family-wise error rate (FWER). The connectivity matrices, alongside fluency scores, were submitted as inputs to the NBS (https://www.nitrc.org/projects/nbs/). A mass univariate approach, using a general linear model, was utilized to discern significant correlations between fluency scores and connectivity strengths across the entire brain, accounting for variations in age, sex and years of education. Upon calculating the test statistic for each connection, we established an initial threshold ranging from 2.0 to 4.0, incremented by 0.1, to identify suprathreshold connections and record their component sizes. To ascertain the significance of these connections, we permuted the order of speech fluency scores and reiterated the analysis 10 000 times to create an empirical null distribution of maximal component sizes. The observed maximal component size was then compared against this null distribution to calculate the *P*-value. Any component with a *P*-value less than 0.1 was deemed significant. The connections comprising such components were recognized as having a significant correlation with the speech fluency scores, thus constituting a network at the whole-brain level. The analyses were executed in MATLAB (version 2019b, MathWorks Inc.), ensuring that the computational environment was consistent and reliable.

Regions pinpointed during the VLSM analysis served as seed regions. Upon identifying networks associated with fluency scores on a whole-brain scale, we elected to showcase the significant connections involving these seed regions and the interconnections with domain-general regions—the latter being those regions interconnected with the seed regions. Visualization of these connections was facilitated by Circos software (http://www.circos.ca/) and the BrainNet Viewer (http://www.nitrc.org/projects/bnv), providing a comprehensive view of the network structure.^26,27^

## Results

### Patients characteristics

The demographic, clinical and behavioural characteristics are shown in Table 1 for the 115 participants included. Mean age was 38.5 years (SD 10.63) and 51.3% were men, mean years of education was 12.55 years (SD 3.55). Years of education was the only demographic variable showing a significant positive correlation with speech fluency (*r* = 0.35). All 115 participants were diagnosed with low-grade glioma (WHO grade II) invading brain areas associated with language. Mean tumour volume was 6.42 × 10^4^ mm^3^ (SD 5.59 × 10^4^) and mean maximum tumour diameter was 68.61 mm (SD 19.19). Neither tumour volume nor maximum tumour diameter was significantly correlated with speech fluency (Supplementary Fig. 4).

**Table 1:** Demographic, clinical and behavioural data of subjects

Demographic, Clinical and Behavioural Data	Patients (*n* = 115)	Correlation with Speech Fluency
Age (years)	38.5 ± 10.63, 19–64	r = −0.15, *P* = 0.10
Sex male/female	59/56	t = −0.08, *P* = 0.94
Education (years)	12.55 ± 3.55, 1–19	r = 0.35, *P* = 0.0001^a^
Handedness right/left	115/0	*n.a.*
Pathological Diagnosis (WHO Grade)	All subjects are patients with glioma (WHO Grade II)	
Lesion/Tumour Volume (mm^3^)	6.42 × 10^4^ ± 5.59 × 10^4^, 1.02 × 10^4^ ± 3.30 × 10^5^	r = −0.06, *P* = 0.55
Tumour maximum Diameter (mm)	68.61 ± 19.19, 32.82–129.45	r = −0.14, *P* = 0.14
MMSE^b^ (%)	89.59 ± 9.06, 47–100	*n.a.*
KPS^b^ (%)	97.13 ± 5.58, 70–100	*n.a.*
Speech Fluency^b^ (%)	95.48 ± 7.52, 70–100	*n.a.*

^a^Age, education, lesion/tumour volumes, tumour maximum diameter and behavioural scores are shown as mean ± standard deviation (SD) and minimum-maximum. Relationships between speech fluency and continuous variables were calculated using Pearson correlation coefficient (r & p). Relationships between speech fluency and categorical data were determined by Student’s t tests (t & p) *: *P* < 0.05.

^b^Behavioural scores have been normalized into percentages based on their full marks. MMSE refers to mini-mental state examination (full mark: 30), KS refers to Karnofsky performance scale (full mark: 100) and speech fluency is acquired from the Chinese version of western aphasia battery test (full mark: 10).

### Domain-specific regions for speech fluency

**Figure 1:**
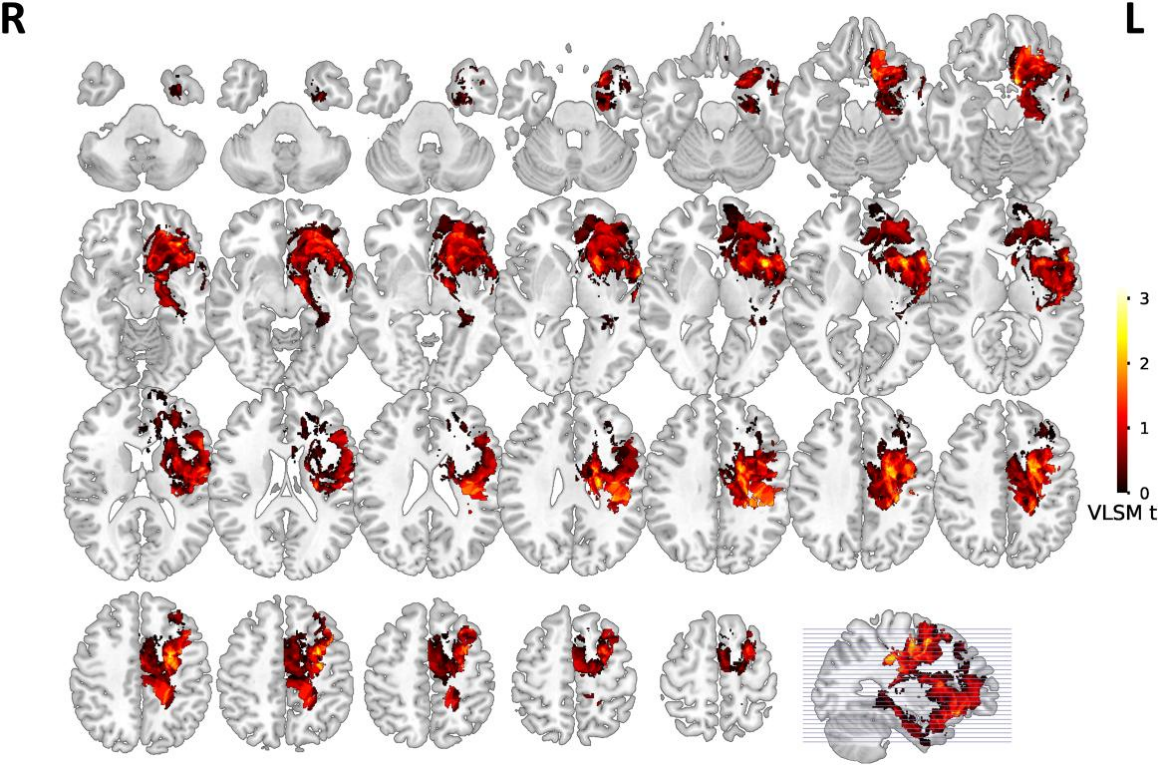
**Voxel-based lesion-symptom mapping for speech fluency in cortical regions**. Representative slices from VLSM maps for speech fluency performance of 115 patients. These maps are colorized depictions of *t*-test results evaluating patient performance on a voxel-by-voxel basis. The warmer the colour is, the lower speech fluency scores of the patients with lesions on the corresponding voxel holding. Age, sex, and education effects have been removed. VLSM, Voxel-based lesion-symptom mapping.

**Figure 2:**
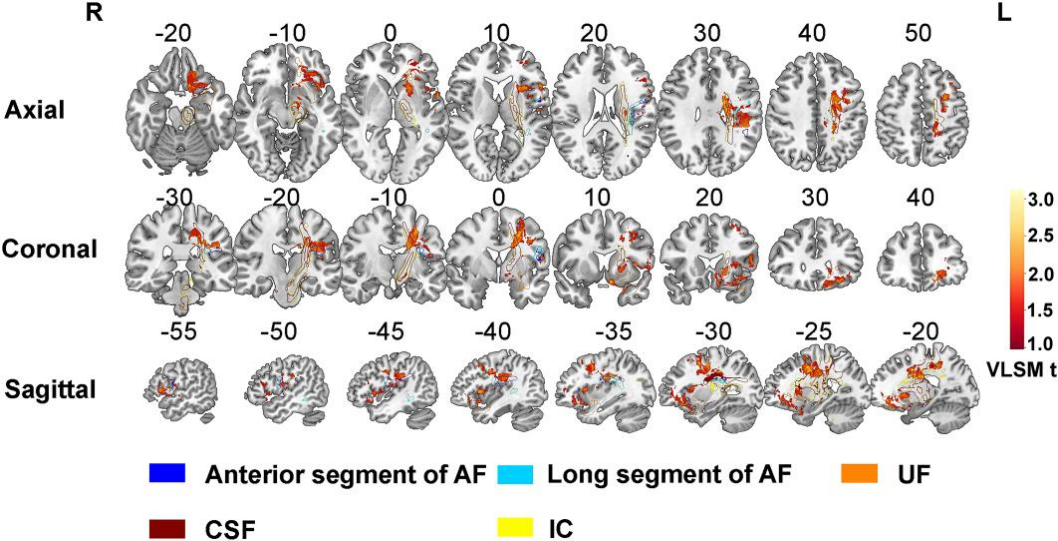
**Voxel-based lesion-symptom mapping for speech fluency in subcortical pathways**. Axial (top), coronal (middle), sagittal (bottom) slices from VLSM maps for speech fluency performance of 115 patients. These maps are colorized depictions of *t*-test results evaluating patient performance on a voxel-by-voxel basis. Higher *t*-value indicates lower speech fluency scores. Age, sex and education effects have been removed. AF = arcuate fasciculus; UF = uncinate fasciculus; IC = internal capsule; CSF = corticospinal fasciculus; VLSM = Voxel-based lesion-symptom mapping.

Across the 115 participants, VLSM maps for speech fluency in cortical regions and white matter pathways are shown in Figs 1 and 2, respectively. Results suggested that there were four cortical regions critical for speech fluency. Based on anatomical criteria, the peak voxels of these regions are in the precentral gyrus (MNI coordinates: *x* = −29, *y* = 1, *z* = 48), the middle frontal gyrus (MNI coordinates: *x* = −30, *y* = 3, *z* = 51), the orbital part of inferior frontal gyrus (MNI coordinates: *x* = −27, *y* = 31, *z* = −16), and the insula (MNI coordinates: *x* = −42, *y* = 13, *z* = 3). To assess white matter tract involvement, we used an atlas-based overlay approach. Catani atlas^28^ were overlaid upon the VLSM map for speech fluency using the Matlab software. To verify the suggested tracts disruption, the tracts were later visually inspected by overlying the VLSM map with the masks of each tract using MRIcron software. Results showed that there were six white matter pathways significantly associated with speech fluency, involving the anterior segment of the arcuate fasciculus (MNI coordinates: *x* = −35, *y* = −15, *z* = 27), the long segment of the arcuate fasciculus (MNI coordinates: *x* = −35, *y* = −15, *z* = 26), the uncinate fasciculus (MNI coordinates: *x* = −8, *y* = 8, *z* = −15), the corticospinal tract (MNI coordinates: *x* = −22, *y* = −2, *z* = 40), the frontal aslant tract (MNI coordinates: *x* = −29, *y* = 1, *z* = 48), and the internal capsule (MNI coordinates: *x* = −19, *y* = −3, *z* = 30) (Table 2). Apart from the insula, domain-specific regions were further validated by intraoperative DCS, with any intraoperative adverse effects (speech arrest) caused by electrical stimulation recorded. We plotted a total of 425 speech arrest sites in Montreal Neurological Institute (MNI) space (Supplementary Fig. 5), and provided the percentage (a proportion of all language sites) of speech arrest sites located in different regions. We also conducted an exact (significance) test and found that the results of DCS and VLSM are highly overlapping (z = 3.33, *P* = 4.35 × 10^−4^). Here, the *P*-value was obtained by exact calculations of risks when DCS and VLSM results do not overlap. The *P*-value was then submitted to determine the value of the z-score through standard normal cumulative distribution.

**Table 2:** MNI coordinates of peak voxels for VLSM results

Brain Region/Fibre Tracts (Left hemisphere)	X	Y	Z	T test
Precentral Gyrus	−29	1	48	3.14
Middle Frontal Gyrus	−30	3	51	2.72
Inferior Frontal Gyrus (Orbital)	−27	31	−16	3.02
Insula	−42	13	3	2.76
Long Segment of Arcuate Fasciculus	−35	−15	27	2.50
Anterior Segment of Arcuate Fasciculus	−35	−15	26	2.50
Uncinate Fasciculus	−8	8	−15	2.69
Corticospinal Tract	−22	−2	40	2.67
Internal Capsule	−19	−3	30	2.72
Frontal Aslant Tract	−25	−2	44	3.04

X, Y and Z refers to MNI coordinates, T test refers to VLSM T test values of peak voxels. All *t*-values are significant (*P* < 0.005). Age, sex and education effects have been removed.

### Brain network of speech fluency

In network analysis, we examined the relationship between connectivity and the behavioural measurement in all subjects. Utilizing the Network-Based Statistic (NBS) across a continuum of primary thresholds (*t* = 2.0–4.0), we identified connections that exhibited significant correlations with fluency scores. Incremental elevation of the primary threshold demonstrated a concomitant decrease in the number of significant connections, as detailed in Supplementary Fig. 6. A threshold of *t* = 2.5, yielding a *P* value of 0.07, delineated a brain network associated with speech fluency at the whole-brain level, ensuring the stability of our results against minor variations.

**Figure 3:**
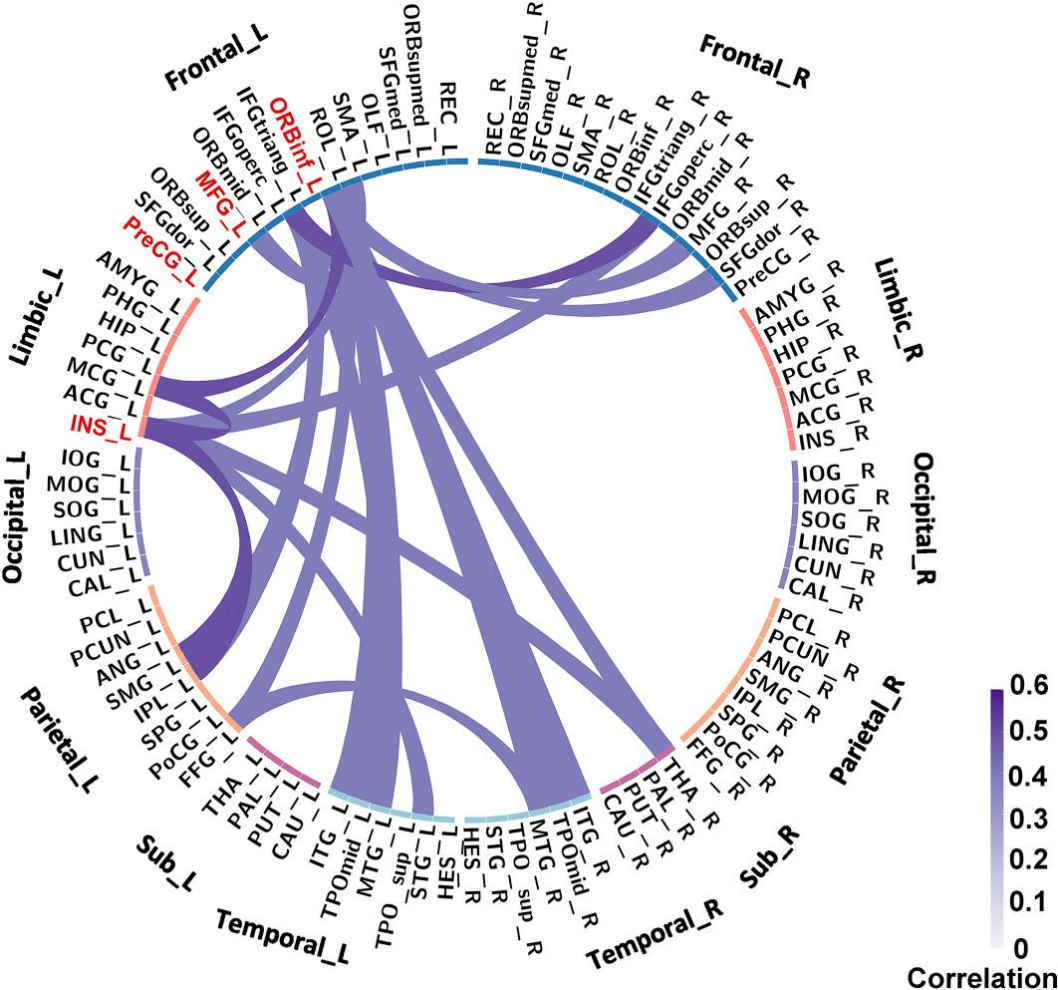
**Connections correlated with speech fluency in glioma patients**. The connections around the seed regions and domain-general regions were obtained from the network at the threshold t = 2.5, which is available in Supplementary Fig. 6. The figure was made using Circos software, 20 nodes and 25 edges were associated with speech fluency in glioma patients. The colour of light purple to purple represents for the partial correlation coefficients (*r*-values) between connections and speech fluency score, with adjusting for age, sex and education effects. The deeper the colour, the stronger the correlation.

**Figure 4:**
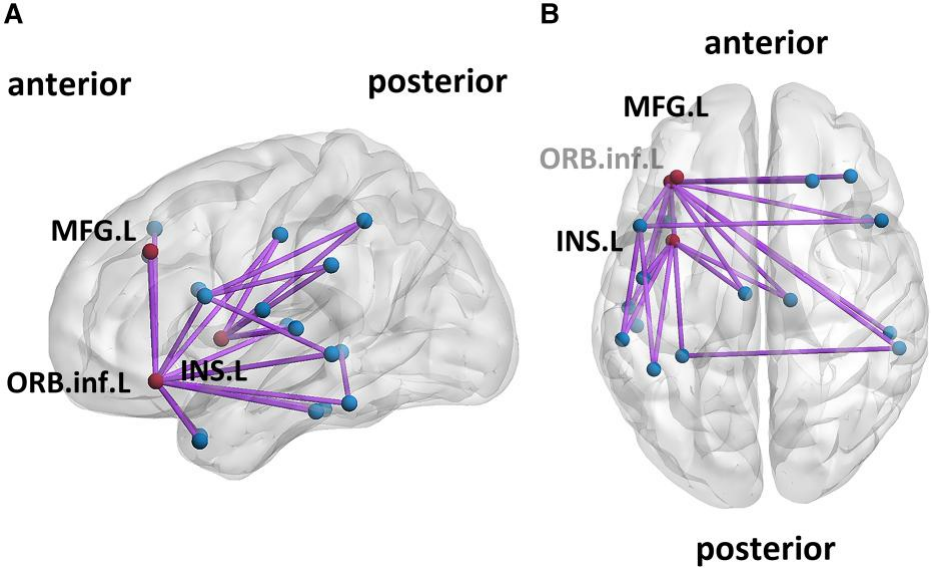
**Connections correlated with speech fluency, lateral and transverse view**. (**A**)The lateral view and (**B**) transverse view of the brain showing the connections around the seed regions (red) and domain-general regions (blue). The figure was made using BrainNet Viewer.

**Figure 5:**
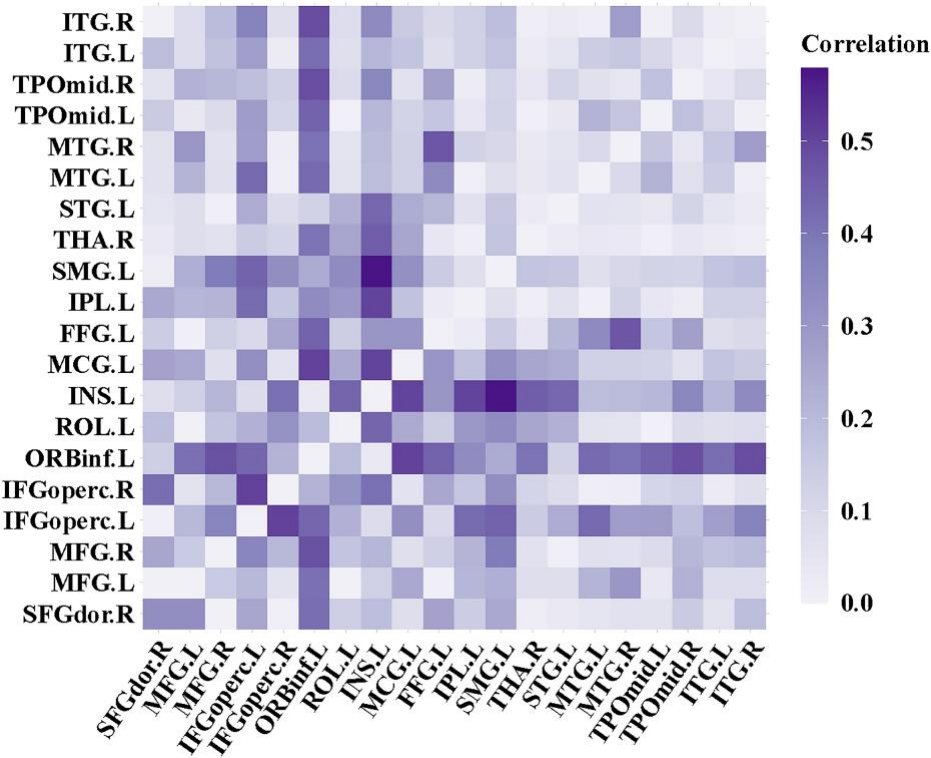
**Heatmap of *r*-values between connections and speech fluency score**. Heatmap of *r*-values between connections and speech fluency score. See Supplementary material for abbreviation list.

We then pinpointed connections leading to the cortical regions identified through the aforementioned VLSM analysis, which contribute to the brain network governing speech fluency. As depicted in Fig. 3, these connections bridge domain-specific regions in the left hemisphere—including the middle frontal gyrus, the inferior frontal gyrus (orbital part), and insula—with 17 brain regions. These regions synergistically interact with domain-specific regions to facilitate fluent speech, conforming to our hypothesis of ‘domain-general regions’. Specifically, five domain-general regions were located within frontal areas, namely the right superior frontal gyrus, right middle frontal gyrus, left and right inferior frontal gyrus (opercular part), and the left rolandic operculum. Additionally, seven domain-general regions were located within temporal areas, left superior temporal gyrus, left and right middle temporal gyrus, left and right temporal pole, left and right inferior temporal gyrus. Other domain-general regions were located in the limbic regions, the parietal regions, and subcortical areas, including left middle cingulate gyrus, left fusiform gyrus, left inferior parietal lobe, left supramarginal gyrus, and right thalamus. Figure 4 illustrates the twenty connections to the domain-specific regions, alongside five inter-domain connections. Notably, five inter-domain connections were observed connecting the (1) left inferior frontal gyrus (opercular part) and right inferior frontal gyrus (opercular part); (2) left inferior frontal gyrus (opercular part) and left inferior parietal lobe; (3) left inferior frontal gyrus (opercular part) and left supramarginal gyrus; (4) left inferior frontal gyrus (opercular part) and left middle temporal gyrus; (5) left fusiform gyrus and right middle temporal gyrus. Figure 5 presents a heatmap delineating the correlation coefficients for all connections among the twenty implicated regions, both domain-specific and domain-general, with speech fluency scores. Definitions and abbreviations for the brain regions involved are enumerated in Supplementary Table 2.

## Discussion

We used multimodal imaging in a relatively large (*n* = 115) cohort of low-grade gliomas to investigate the brain network of speech fluency. VLSM analysis found six white matter pathways and four domain-specific regions for speech fluency. Domain-specific regions include the precentral gyrus, the middle frontal gyrus, the inferior frontal gyrus, and the insula. Apart from the insula, the domain-specific regions were validated by direct cortical stimulation (DCS) intra-operatively. Connectivity-behaviour analyses revealed that the domain-specific regions are part of a network that extends to regions in the temporal, frontal, parietal, limbic lobes, as well as subcortical regions. These regions functionally interact with the domain-specific regions during the speech process. The extent of the above network supports our hypothesis that fluent speech engages a network consisting of two components: the ‘domain-specific regions’ and the ‘domain-general’ regions.

One implication of the current finding is that the inferior frontal gyrus (BA47) and its underlying white matter pathways were associated with speech fluency, perhaps by supporting semantic subprocesses during fluent speech. Although the left BA44 and BA45 (classic Broca’s area) have been regarded as classic cortical language areas,^29,30^ BA47 may play a central role in speech production. A meta-analysis of fMRI studies in native speakers of English suggested that BA47 was simultaneously activated during speech production with other inferior frontal areas, and it has a role in semantic aspects of language.^31^ The current study pinpoints the importance of BA47 and verifies its central role in speech production using VLSM analysis in native Chinese speakers. In addition, the current study found the uncinate fasciculus, i.e. the white matter structural connection underlying BA47, associated with speech fluency. The uncinate fasciculus (UF) structurally connects BA47 to the parts of limbic system.^32^ Using functional connectivity analysis, we also found that the inferior frontal gyrus (orbital part) and the temporal pole were functionally connected. Therefore, the consistent result from structural and functional connectivity provides strong evidence that the UF underlying the inferior frontal gyrus (BA47) participates in fluent speech production.

In addition to the UF, results suggested that other white matter pathways underlying the posterior inferior frontal gyrus such as the arcuate fasciculus (AF) are related to fluent speech production. A previous study suggested that impairment of speech production can be explained by co-occurring damage of white matter pathways in the vicinity of the anterior segment of the AF.^33^ However, concurrent damage to white matter pathways as the consequence of stroke hindered their dissociation. Low-grade glioma widely distributed across the brain. This distributed pattern allows us to further identify the white matter pathways in the vicinity of the anterior AF. In the current study, lesion-symptom mapping analysis in gliomas patients found white matter pathways in addition to the anterior AF, long segment of AF and the frontal aslant tract (FAT) should also be included in the language network. First, the anterior segment of AF connects Broca’s area with the parietal cortex, and the long segment of AF connects Broca’s area with the temporal cortex.^34^ Our findings indicated that maintenance of speech flow not only relies on the classic anterior areas (the inferior frontal gyrus) but also the posterior areas. Second, the FAT is a direct pathway connecting the supplementary motor areas and the inferior frontal lobe,^35^ this white matter may exerts a role in the integration of the regions within the frontal lobe.

The left precentral gyrus (PreCG) and its underlying white matter regions including the corticospinal fasciculus and internal capsule were associated with speech fluency. Both MRI and lesion studies have reported that the dorsal middle part of the left PreCG was found to be associated with apraxia of speech (AOS), which is a motor speech disorder.^36-39^ Therefore, the disrupted speech fluency caused by damaged PreCG may be due to other underlying diagnoses besides aphasia, such as AOS. In terms of the white matter underlying the left PreCG, the corticospinal fasciculus and internal capsule are involved in controlling the physical movements of the tongue, lips and larynx,^40,41^ with impaired speech muscle movements naturally resulting in the reduction of speech fluency. Just in front of the left PreCG, the left middle frontal gyrus (MFG) was also found associated with speech fluency performance, at least in native Chinese speakers. The above finding also concurred with our DCS results. A previous study in non-alphabetic populations has reported that MFG plays a role in phonological processing. For example, a meta-analysis suggested that the MFG is important for phonological processing in overt Chinese reading.^42^ A study in another non-alphabetic language (Japanese) showed that the MFG was activated in the processing of phonological manipulation.^43^ In the current study, speech fluency tasks require individuals to formulate the correct phonological representation. Therefore, the involvement of MFG in speech fluency could be explained by phonological processing. Taken together, these findings indicate that the left MFG is involved in the production of fluent speech by phonological processing before output to the left PreCG and its underlying white matter pathways (the motor articulatory system), enabling thoughts to be turned into words, phrases, and sentences. An alternative explanation for our findings of the left MFG and the left PreCG is the language functional reorganization in these two regions. A recent DCS study contrasted the cortical mappings obtained during two brain resections and reported a topological migration of language sites within the left the dorsolateral prefrontal cortex (lies in MFG) and the left ventral premotor cortex (lies in PreCG), which has a relatively high potential for evolving reorganizations.^44^

Our network analysis has elucidated that domain-specific regions are integral components of a densely interconnected network, which encompasses domain-general regions across cortical and subcortical areas, such as the middle frontal gyrus, rolandic operculum, superior temporal gyrus, temporal pole, middle temporal gyrus, inferior temporal gyrus, middle cingulate gyrus, inferior parietal lobe, fusiform gyrus, supramarginal gyrus, and the thalamus. Some of these domain-general regions (superior temporal gyrus, temporal pole, middle temporal gyrus) have previously reported to be involved in language processing.^45-47^ For example, it was concluded from a meta-analysis of fMRI studies that the left middle temporal gyrus specialized for semantic processing of Chinese characters.^45^ Collectively, these domain-general regions are hypothesized to form distinct networks that underpin the fluency of speech. Notably, the middle frontal gyrus, along with the inferior parietal lobe, may constitute the fronto-parietal networks (FPN), which is also thought to be conducive to speech fluency.^48^ According to Cabeza *et al*., the right FPN is believed to engage in monitoring functions, whereas the left FPN appears to play a critical role in production processes such as speech output.^49^ Among the interconnections between domain-specific and domain-general regions, connections of left insula and left supramarginal gyrus showed the strongest correlation to speech fluency, which may be explained by the structural connectivity between these regions.^50^ Moreover, the domain-general regions, such as the temporal pole, supramarginal gyrus, thalamus, rolandic operculum, have been previously implicated in speech production mechanisms.^46,51-56^ Research led by Mesulam *et al*. suggests that impairments in the temporopolar region are associated with semantic variants of primary progressive aphasia and semantic dementia.^46^ Our data lend additional support to the pivotal role of the left temporal pole in linguistic functions, aligning with these established frameworks. Additionally, we found that the middle cingulate gyrus is associated with speech fluency performance. Previous studies emphasized the role of the anterior cingulate gyrus in attentional control of language,^57^ while the current finding of middle cingulate gyrus may be explained by involvement in skeletal muscle movement regulation.^58^ Our study not only delineates the cortical regions involved in speech fluency but also corroborates these findings with the identification of underlying white matter tracts, which form the structural framework for such functional connectivity. For example, the uncinate fasciculus, as we have observed, bridges the temporal pole with the orbitofrontal cortex. The frontal aslant tract, connecting the Broca’s area with the supplementary motor area and the anterior cingulate cortex, is thought to mediate speech initiation and suppression, thus contributing to fluent speech regulation, as we previously discussed.

We recognize that functional reorganization, potentially triggered by tumour growth, may commence preoperatively, facilitating the redistribution of language functions to viable brain regions.^59,60^ We were unable to examine the effect of functional reorganization to the brain network of speech fluency in the current study. Based on evidence from previous studies in patients with glioma, functional reorganization occurs usually in hierarchical order by first recruiting perilesional regions and intralesional regions and later contralesional regions for compensation.^61,62^ In addition to functional regions, slow-growing tumours also affect the local and global functional connectivity between functional regions. Our team recently investigated the network reorganization in preoperative patients with gliomas (mainly low-grade glioma) involving language network, results indicated slow-grow tumours disrupted the local and global functional interactions in patients, and disruptions were significantly related to deficit-severity.^63^

In addition, our study has several limitations. First, the WAB-R fluency scale uses multidimensional 10-point rating system that integrates across many subcomponents (e.g. grammaticality, lexical retrieval, length) of connected speech to assess the degree of fluency.^4^ Here, we aim to gain a holistic understanding of the speech fluency network, future studies may collect behavioural data based on articulatory and grammatical sub-components and disentangle their contributions to the speech fluency. Second, our study could not provide evidence for causal interactions between each cortical regions on time scales. In future work, direct intracranial cortical recordings such as electrocorticography (ECoG) with high temporal resolution should be applied to capture which cortical areas interact with one another during fluent speech processing, providing more insight into the dynamics of the language networks. Moreover, we were unable to stimulate insular cortex, because the insula is hidden deep within the lateral sulcus and not visible from the surface view, future study may employ deep electrode implanted in the insular cortex to verify its contribution to speech fluency.

In summary, we investigated the brain network of speech fluency in Chinese native speakers. Results indicated that speech fluency engages a bilateral network supported by domain-specific regions, domain-general regions, and white matter pathways. Domain-specific regions apart from insula were validated by intra-operative direct cortical stimulation. The domain-general regions functionally interacted with the domain-specific regions when fluent speech occurs. Our findings underlie the previous idea of two-component brain network, which includes the ‘domain-specific regions’ and ‘domain-general’ regions. Our delineation of speech fluency network may also help clinicians to locate and preserve language function during brain tumour resection.

## Supplementary Material

fcae153_Supplementary_Data

## Data Availability

The data that support the findings of this study are available on request from the corresponding author. The data are not publicly available due to privacy or ethical restrictions.
