# Supplementary material for: A speech fluency brain network derived from gliomas
Source: Brain Commun. 2024 Apr 29;6(3):fcae153. doi: 10.1093/braincomms/fcae153 (PMC11098038; doi:10.1093/braincomms/fcae153)
Supplement: fcae153_Supplementary_Data [file fcae153_supplementary_data.docx]

**Supplementary Table 1 The scoring criteria of ABC fluency scale**

| **Scoring Criteria** | **score** |
| --- | --- |
| No words or short, meaningless utterances. | 0 |
| Recurrent stereotypic utterance with varied intonation, conveying some meaning. | 1 |
| Single word, often paraphasias, effortful and hesitant. | 2 |
| Fluent recurrent utterances or mumbling, very low volume jargon. | 3 |
| Halting, telegraphic speech. Mostly single word, often paraphasic but with occasional verbs or prepositional phrases. Automatic sentences only, e.g., “Oh I don’t know.” | 4 |
| Often telegraphic but more fluent speech with some grammatical organization. Paraphasias may be prominent. Few propositional sentences. | 5 |
| More complete propositional sentences. Normal syntactic pattern may be present. Paraphasias may be present. | 6 |
| Phonemic jargon with semblance to English syntax and rhythm with varied phonemes and neologisms. May be voluble; must be fluent. | 7 |
| Circumlocutory, fluent speech. Marked word finding difficulty. Verbal paraphasias. May have semantic jargon. The sentences are often complete but may be irrelevant. | 8 |
| Mostly complete, relevant sentences; occasional hesitation and/or paraphasias. Word finding difficulty. May have some articulatory errors. | 9 |
| Sentences of normal length and complexity, without definite slowing, halting, or articulatory difficulty. No paraphasias. | 10 |

**Supplementary Table 2 Abbreviation of 90 anatomical regions in the automated anatomical labelling (AAL) atlas**

| **No.** | **Abbreviation** | **Anatomical Description** |
| --- | --- | --- |
| 1 | PreCG.L | Precental gyrus |
| 2 | PreCG.R | Precental gyrus |
| 3 | SFGdor.L | Superior frontal gyrus, dorsolateral |
| 4 | SFGdor.R | Superior frontal gyrus, dorsolateral |
| 5 | ORBsup.L | Superior frontal gyrus, orbital part |
| 6 | ORBsup.R | Superior frontal gyrus, orbital part |
| 7 | MFG.L | Middle frontal gyrus |
| 8 | MFG.R | Middle frontal gyrus |
| 9 | ORBmid.L | Middle frontal gyrus, orbital part |
| 10 | ORBmid.R | Middle frontal gyrus, orbital part |
| 11 | IFGoperc.L | Inferior frontal gyrus, opercular part |
| 12 | IFGoperc.R | Inferior frontal gyrus, opercular part |
| 13 | IFGtriang.L | Inferior frontal gyrus, triangular part |
| 14 | IFGtriang.R | Inferior frontal gyrus, triangular part |
| 15 | ORBinf.L | Inferior frontal gyrus, orbital part |
| 16 | ORBinf.R | Inferior frontal gyrus, orbital part |
| 17 | ROL.L | Rolandic operculum |
| 18 | ROL.R | Rolandic operculum |
| 19 | SMA.L | Supplementary motor area |
| 20 | SMA.R | Supplementary motor area |
| 21 | OLF.L | Olfactory cortex |
| 22 | OLF.R | Olfactory cortex |
| 23 | SFGmed.L | Superior frontal gyrus, medial |
| 24 | SFGmed.R | Superior frontal gyrus, medial |
| 25 | ORBsupmed.L | Superior frontal gyrus, medial orbital |
| 26 | ORBsupmed.R | Superior frontal gyrus, medial orbital |
| 27 | REC.L | Gyrus rectus |
| 28 | REC.R | Gyrus rectus |
| 29 | INS.L | Insula |
| 30 | INS.R | Insula |
| 31 | ACG.L | Anterior cingulate and paracingulate gyri |
| 32 | ACG.R | Anterior cingulate and paracingulate gyri |
| 33 | MCG.L | Median cingulate and paracingulate gyri |
| 34 | MCG.R | Median cingulate and paracingulate gyri |
| 35 | PCG.L | Posterior cingulate gyrus |
| 36 | PCG.R | Posterior cingulate gyrus |
| 37 | HIP.L | Hippocampus |
| 38 | HIP.R | Hippocampus |
| 39 | PHG.L | Parahippocampal gyrus |
| 40 | PHG.R | Parahippocampal gyrus |
| 41 | AMYG.L | Amygdala |
| 42 | AMYG.R | Amygdala |
| 43 | CAL.L | Calcarine fissure and surrounding cortex |
| 44 | CAL.R | Calcarine fissure and surrounding cortex |
| 45 | CUN.L | Cuneus |
| 46 | CUN.R | Cuneus |
| 47 | LING.L | Lingual gyrus |
| 48 | LING.R | Lingual gyrus |
| 49 | SOG.L | Superior occipital gyrus |
| 50 | SOG.R | Superior occipital gyrus |
| 51 | MOG.L | Middle occipital gyrus |
| 52 | MOG.R | Middle occipital gyrus |
| 53 | IOG.L | Inferior occipital gyrus |
| 54 | IOG.R | Inferior occipital gyrus |
| 55 | FFG.L | Fusiform gyrus |
| 56 | FFG.R | Fusiform gyrus |
| 57 | PoCG.L | Postcentral gyrus |
| 58 | PoCG.R | Postcentral gyrus |
| 59 | SPG.L | Superior parietal gyrus |
| 60 | SPG.R | Superior parietal gyrus |
| 61 | IPL.L | Inferior parietal, but supramarginal and angular gyri |
| 62 | IPL.R | Inferior parietal, but supramarginal and angular gyri |
| 63 | SMG.L | Supramarginal gyrus |
| 64 | SMG.R | Supramarginal gyrus |
| 65 | ANG.L | Angular gyrus |
| 66 | ANG.R | Angular gyrus |
| 67 | PCUN.L | Precuneus |
| 68 | PCUN.R | Precuneus |
| 69 | PCL.L | Paracentral lobule |
| 70 | PCL.R | Paracentral lobule |
| 71 | CAU.L | Caudate nucleus |
| 72 | CAU.R | Caudate nucleus |
| 73 | PUT.L | Lenticular nucleus, putamen |
| 74 | PUT.R | Lenticular nucleus, putamen |
| 75 | PAL.L | Lenticular nucleus, pallidum |
| 76 | PAL.R | Lenticular nucleus, pallidum |
| 77 | THA.L | Thalamus |
| 78 | THA.R | Thalamus |
| 79 | HES.L | Heschl gyrus |
| 80 | HES.R | Heschl gyrus |
| 81 | STG.L | Superior temporal gyrus |
| 82 | STG.R | Superior temporal gyrus |
| 83 | TPOsup.L | Temporal pole: superior temporal gyrus |
| 84 | TPOsup.R | Temporal pole: superior temporal gyrus |
| 85 | MTG.L | Middle temporal gyrus |
| 86 | MTG.R | Middle temporal gyrus |
| 87 | TPOmid.L | Temporal pole: middle temporal gyrus |
| 88 | TPOmid.R | Temporal pole: middle temporal gyrus |
| 89 | ITG.L | Inferior temporal gyrus |
| 90 | ITG.R | Inferior temporal gyrus |


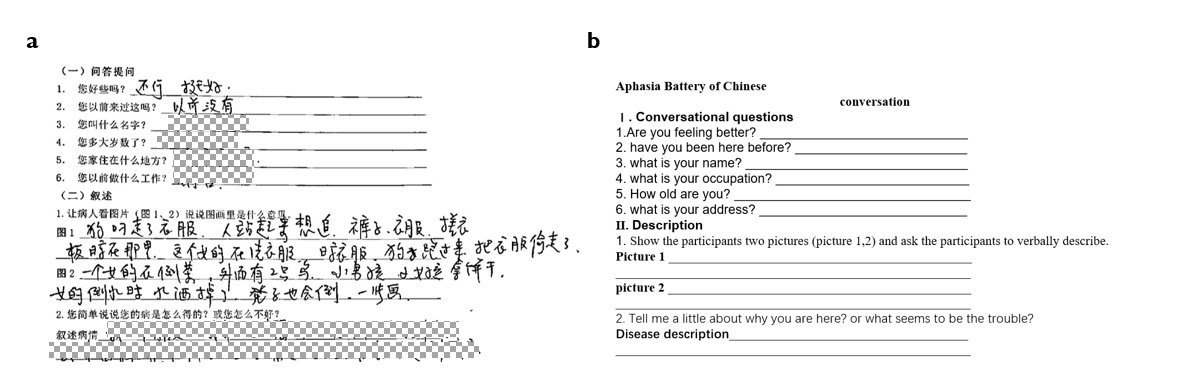
**Supplementary Figure 1 Speech fluency tasks in Aphasia Battery of Chinese**

**(a)** Speech fluency task includes conversational questions and description. In Conversational questions, participants need to verbally respond to six routine questions (e.g., questions such as “Are you feeling better?”, “have you been here before?”, “what is your name?”, “what is your occupation?”, “How old are you?” and “what is your address?”). Description including two subtasks: picture description and disease description. In picture description, the examiner presents two pictures and say: “tell me what you see. Try to talk in sentences.” In disease description, the examiner asks one open-ended question: “tell me a little about why you are here? or what seems to be the trouble?”.

**(b)** The English version of speech fluency tasks in Aphasia Battery of Chinese.


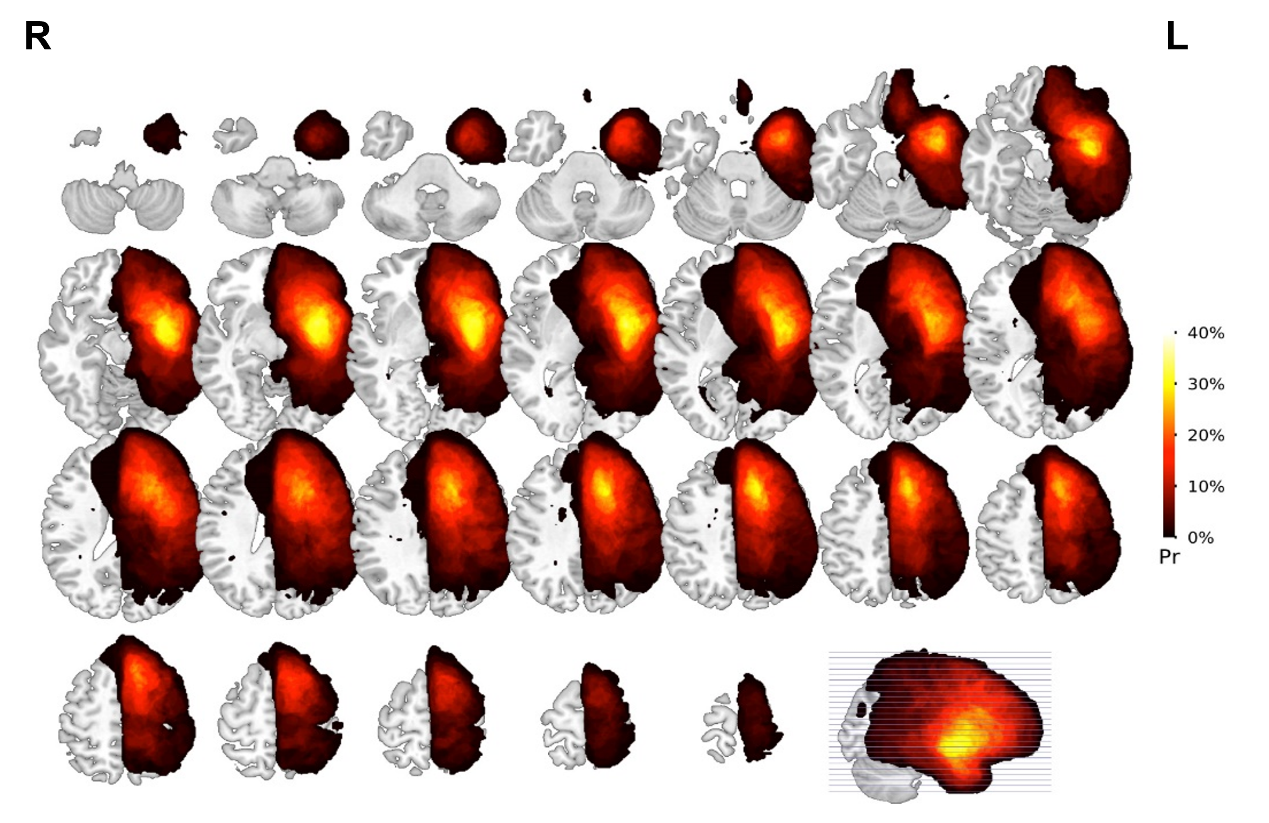


**Supplementary Figure 2 Topography of Tumor Distribution**

Topography is shown using a heat map. The hotter the color is, tumors of more patients are on the voxel (color bar indicates proportional overlap). Pr: Proprotion.


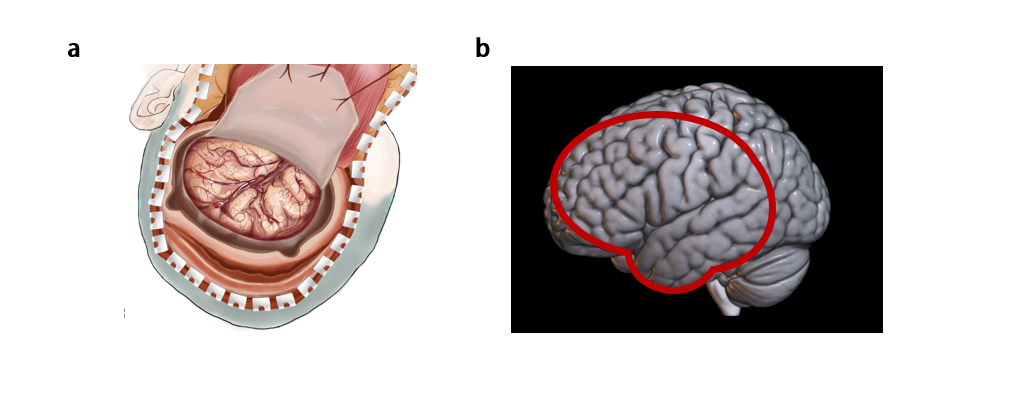


**Supplementary Figure 3 The exposed cortex in direct cortical stimulation**

a) Schematic diagram of frontotemporal craniotomy. b) All patients (n=115) had the same range of exposed cortex (circled in red line), which involves the frontal and temporal lobes, as well as a portion of the parietal lobe. The exposed cortex was stimulated at an interval of 1 cm during language mapping.


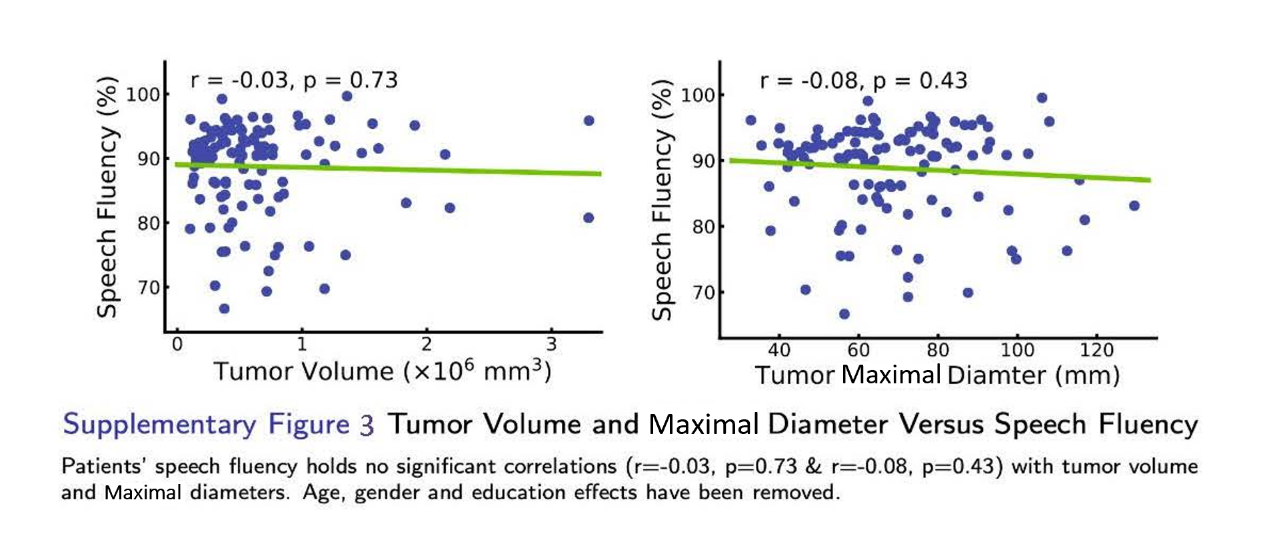


**Supplementary Figure 4** **Correlations between speech fluency and tumour volume as well as tumour maximal diameter**

The results indicate there is no statistical significance between speech fluency and tumor volume (partial correlation coefficient=-0.03, p=0.73, sample size=115), as well as maximal diameters (partial correlation coefficient=-0.08, p=0.43, sample size=115) with adjusting for age, gender and education effects.


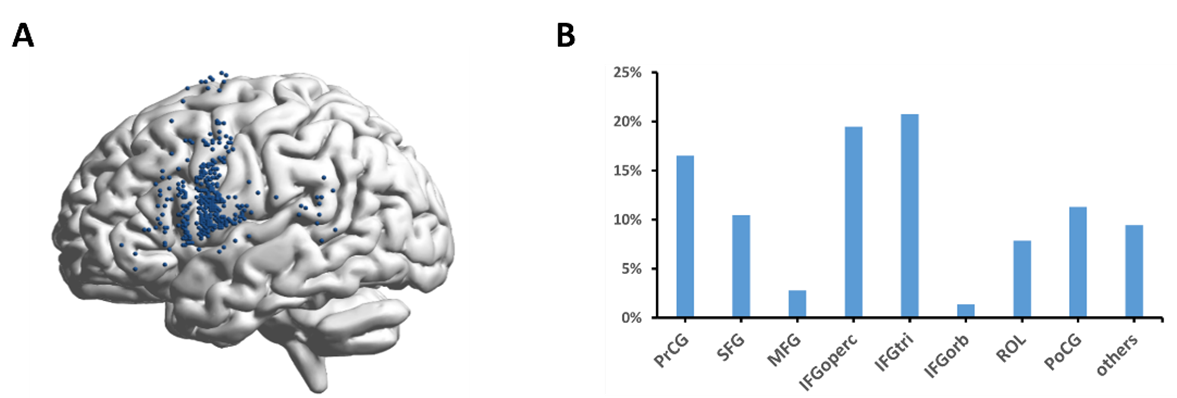


**Supplementary Figure 5 Spatial distribution of speech arrest sites**

(A) A total of 425 speech arrest sites from all patients (n=115) were plotted in the MNI space. (B) The percentage of speech arrest sites in the different regions is shown. Apart from insula, brain regions (PrCG, MFG, and IFGorb) resulting from VLSM analysis were validated by direct cortical stimulation. PrCG = precentral gyrus; SFG = superior frontal gyrus; MFG = middle frontal gyrus; IFGoperc = inferior frontal gyrus, opercular part; IFGtri = inferior frontal gyrus, triangular part; IFGorb = inferior frontal gyrus, orbital part; ROL = rolandic operculum; PoCG = postcentral gyrus.


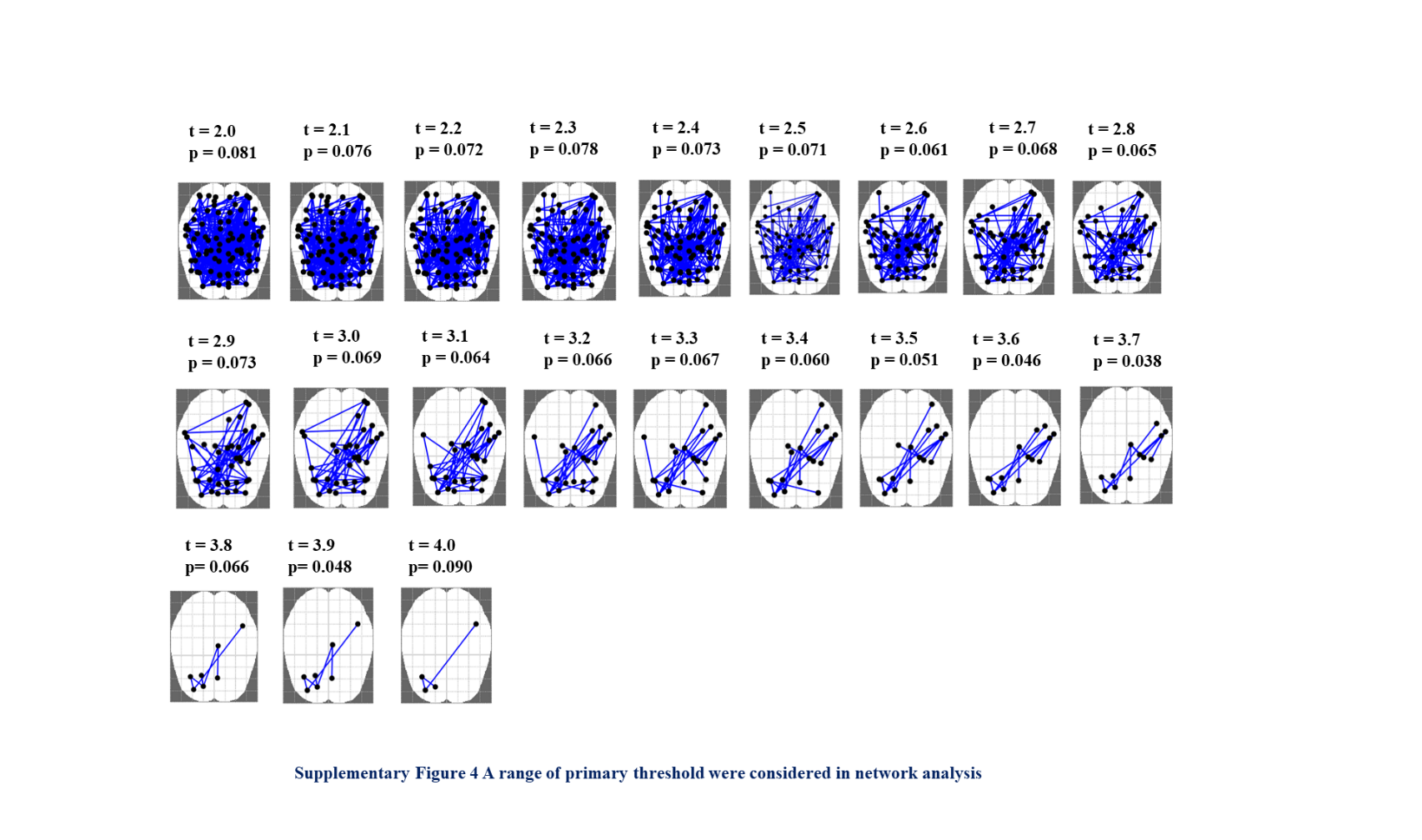


**Supplementary Figure 6** **Results of NBS at a range of different thresholds.**

The NBS identified subnetworks comprising edges associated with speech fluency under different primary thresholds (t-value). The NBS also provide the p-value for corresponding network. NBS: Network-Based Statistic.
